# Human Vimentin Layers on Solid Substrates: Adsorption Kinetics and Corona Formation Investigations

**DOI:** 10.1021/acs.biomac.2c00415

**Published:** 2022-07-13

**Authors:** Monika Wasilewska, Paulina Żeliszewska, Katarzyna Pogoda, Piotr Deptuła, Robert Bucki, Zbigniew Adamczyk

**Affiliations:** † Jerzy Haber Institute of Catalysis and Surface Chemistry, Polish Academy of Sciences, PL-30239 Krakow, Poland; ‡ Institute of Nuclear Physics, Polish Academy of Sciences, PL-31342 Krakow, Poland; § Department of Medical Microbiology and Nanobiomedical Engineering, Medical University of Białystok, PL-15222 Białystok, Poland

## Abstract

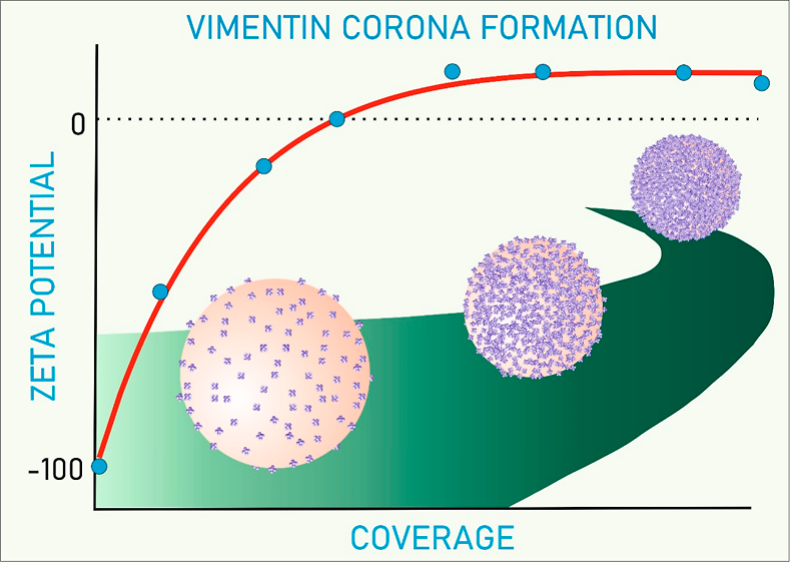

Adsorption kinetics of human vimentin on negatively charged substrates (mica, silica, and polymer particles) was analyzed using atomic force microscopy (AFM), quartz microbalance (QCM), and the laser doppler velocimetry (LDV) method. AFM studies realized under diffusion conditions proved that the adsorbed protein layer mainly consisted of aggregates in the form of compact tetramers and hexamers of a size equal to 11–12 nm. These results were consistent with vimentin adsorption kinetics under flow conditions investigated by QCM. It was established that vimentin aggregates efficiently adsorbed on the negatively charged silica sensor at pH 3.5 and 7.4, forming compact layers with the coverage reaching 3.5 mg m^–2^. Additionally, the formation of the vimentin corona at polymer particles was examined using the LDV method and interpreted in terms of the electrokinetic model. This allowed us to determine the zeta potential of the corona as a function of pH and the electrokinetic charge of aggregates, which was equal to −0.7 e nm^–2^ at pH 7.4 in a 10 mM NaCl solution. The anomalous adsorption of aggregates exhibiting an average negative charge on the negatively charged substrates was interpreted as a result of a heterogeneous charge distribution. These investigations confirmed that it is feasible to deposit stable vimentin layers both at planar substrates and at carrier particles with well-controlled coverage and zeta potential. They can be used for investigations of vimentin interactions with various ligands including receptors of the innate immune system, immunoglobulins, bacterial virulence factors, and spike proteins of viruses.

## Introduction

Vimentin is a member of the family of intermediate filament proteins, which are the constituents of highly ordered cell fiber networks.^1^ It is a crucial component of the cytoskeleton that assures a proper architecture of cells and mechanical defense against extracellular stress. Vimentin participates in cellular processes linked with numerous diseases, for example, cataract formation (vimentin filaments have an essential role in maintaining the lens morphology and integrity),^2^ inflammation (rheumatoid arthritis and Crohn’s disease)^3,4^ and different cancers.^5−8^ It also plays an essential role in wound healing.^9^ Likewise, it mediates the activation of a number of signaling pathways.^10^ It is also involved in cell infections by RNA and DNA viruses. Reference (11) reports a significant role of vimentin in SARS-CoV virus entry through interaction with its spike protein. Therefore, it was classified as a cellular factor prevalent in the SARS-CoV spike protein-ACE2 complexes, suggesting that it may serve as a target for antiviral drugs.

As far as the basic structure is concerned, monomeric vimentin consists of a single 464 amino acid chain, mostly alpha-helical,^12^ which is split into three coiled-coil sections (77 amino acid “head,” a 326-residue “rod,” and a 61-residue “tail”).^12−14^ The molar mass of monomeric vimentin equals 53.5 kg mol^–1^ (kDa).^15^ However, despite its essential significance, little is known about the basic physicochemical properties of the molecule, which is mainly due to the fact that, unlike actin and tubulin, it is resistant to crystallization, except for small fragments.^16^ Therefore, adequate molecular dynamics modeling considering the molecule folding was not performed. In consequence, the molecule size, conformations, and charge as a function of pH were not theoretically predicted.

On the other hand, the experimental investigations are mainly focused on vimentin assembly mechanisms and kinetics. In a simplified model of vimentin organization, two monomers create parallel coiled coils and then two dimers build a tetramer by antiparallel dimerization. It is assumed that the tetramer length equals to 60 nm and the diameter equals to 5 nm.^17,18^ Such tetramers remain stable in low-salt buffers. Afterward, unit-length filaments (ULFs) are created by the lateral assembly of eight tetramers in longitudinal annealing and compaction processes having the diameter of 11 nm.^19^ In cells vimentin molecules form various structures ranging from soluble tetramers to the filaments^20,21^ Vimentin molecule assembly can be triggered in vitro by the presence of KCl or NaCl, and this process has been extensively studied.^22−26^

However, despite the significance of vimentin biological functions and key role in many cellular processes, there is scarce information in the literature concerning its basic properties and adsorption kinetics for solid supports. Because of the deficit of such data, the main objective of this paper was to acquire comprehensive characteristics of vimentin molecule aggregates comprising their electrokinetic properties and to elucidate mechanisms of their adsorption at planar substrates such as mica or silica. The results obtained in such model systems are complemented by investigations of vimentin corona formation at polymer carrier particles using in situ electrokinetic techniques. The newly acquired knowledge is used to develop efficient procedures for the preparation of stable vimentin monolayers in solid substrates of well-defined coverage, molecule orientation, and charge distribution. It is argued that such layers can be exploited in systematic studies focused on determining vimentin interactions with macroion ligands comprising immunoglobulins or spike proteins of SARS-CoV-2.

## Materials and Methods

All chemicals used in experiments were reagents of analytical grade and were used without any extra purification. His-tagged recombinant human vimentin (lyophilized from sterile 40% acetonitrile, 0.1% TFA) was purchased from Sino Biological (10028-H08B). The stock suspension was reduced to a concentration of 0.1–5 mg L^–1^ before each experiment.

Sodium chloride (NaCl) and phosphate-buffered saline (PBS) were commercial products of Sigma-Aldrich. Other chemical reagents (NaOH, HCl, etc.) were obtained from Avantor Performance Materials Poland S.A. Distilled water was obtained using a Milli-Q Elix&Simplicity 185 purification system from Millipore.

The ionic strength of vimentin solutions was adjusted by the addition of sodium chloride, and the pH of the solution was regulated by the addition of hydrochloric acid (3.5–4), PBS (pH 7.4), and sodium hydroxide (pH 8–10).

The mica plates supplied from Continental Trade were used as a solid support for vimentin adsorption. The thin samples of mica were directly cleaved before each experiment. Additionally, in vimentin corona formation experiments, sulfonate polystyrene particles (negatively charged and referred to as S800), products synthesized in our laboratory in accordance with the Goodwin procedure,^27^ served as colloid carriers.

The electrophoretic mobility of the microparticles, without and with the protein corona, was determined using the laser Doppler velocimetry (LDV) using the Zetasizer Nano ZS instrument from Malvern.

The quartz microbalance (QCM) was used to determine the kinetics of vimentin adsorption. The gravimetric measurements were performed with the use of a Q-Sense E1 system (Q-Sense, Gothenburg, Sweden) in accordance with the standard protocol described in ref (28). The experimental runs were initiated after reaching a stable baseline for the pure electrolyte. The flow of the electrolyte was set up for 2.5 × 10^–3^ cm^3^ s^–1^. Subsequently, the vimentin solution with the desired concentration (typically 5 mg L^–1^) was passed through the cell. After attaining a desired coverage, the protein layer was flushed with the pure electrolyte in order to determine its stability. In order to calculate the adsorbed vimentin mass per unit area (coverage), the Sauerbrey equation was used^29^
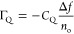
1where Γ_Q_ is the mass coverage, *C*_Q_ is the mass (coverage) sensitivity constant equal to 0.177 mg m^–2^ Hz^–1^ for the 5 MHz AT-cut quartz sensor, Δ*f* is the frequency change, and *n*_o_ is the overtone number.

In the AFM study, the mica sheets were submerged in a low concentration solution of vimentin (0.5 mg L^–1^). The adsorption process took place under thermostated conditions under the diffusion-controlled transport for 10–180 min. The mica sheets with adsorbed molecules were rinsed using ultrapure water for 30 s. The vimentin layers were subsequently imaged using the NT-MDT Solver AFM with SMENA—B scanning head working in air. The silicon AFM probes (polysilicon cantilevers, ETALON) with nominal resonance frequencies ranging from 77 to 114 kHz and a radius of curvature equal to 10 nm were used. Randomly chosen scan areas of 2.0 μm × 2.0 μm were imaged over each sample with up to 20 different locations, reaching the relative precision of 5%. The temperature through the measurements was constant and kept to be equal to 298 K.

### Characteristics of Substrates

Thorough physicochemical characteristics of mica and silica sensors used in investigations of vimentin adsorption kinetics were carried out using AFM (surface topography) and electrokinetic measurements (for details consult the Supporting Information section). Thus, the rms for mica sheets determined by AFM was below or equal to 0.1 nm, whereas for the QCM sensors, it was equal to 0.8 nm.

Detailed characterizations of the polymer particles used as carriers in the vimentin corona formation experiments were performed using DLS (diffusion coefficient) and the LDV measurements (electrophoretic mobility). Using these experimental data, the hydrodynamic diameter of particles was calculated using the Stokes–Einstein formula and the zeta potential using the Smoluchowski–Henry relationship. Independently, laser diffractometry and AFM imaging were used to determine the size of the particles. It was equal to 820 ± 20 nm. The zeta potential of particles was equal to −70 and −90 mV at pH 3.5 and 7.4, respectively (for an ionic strength of 0.01 M). Dependencies of the zeta potential of these polymer carriers on pH are graphically shown in the Supporting Information.

## Results and Discussion

### Vimentin Adsorption Kinetics—AFM Measurements

**Figure 1:**
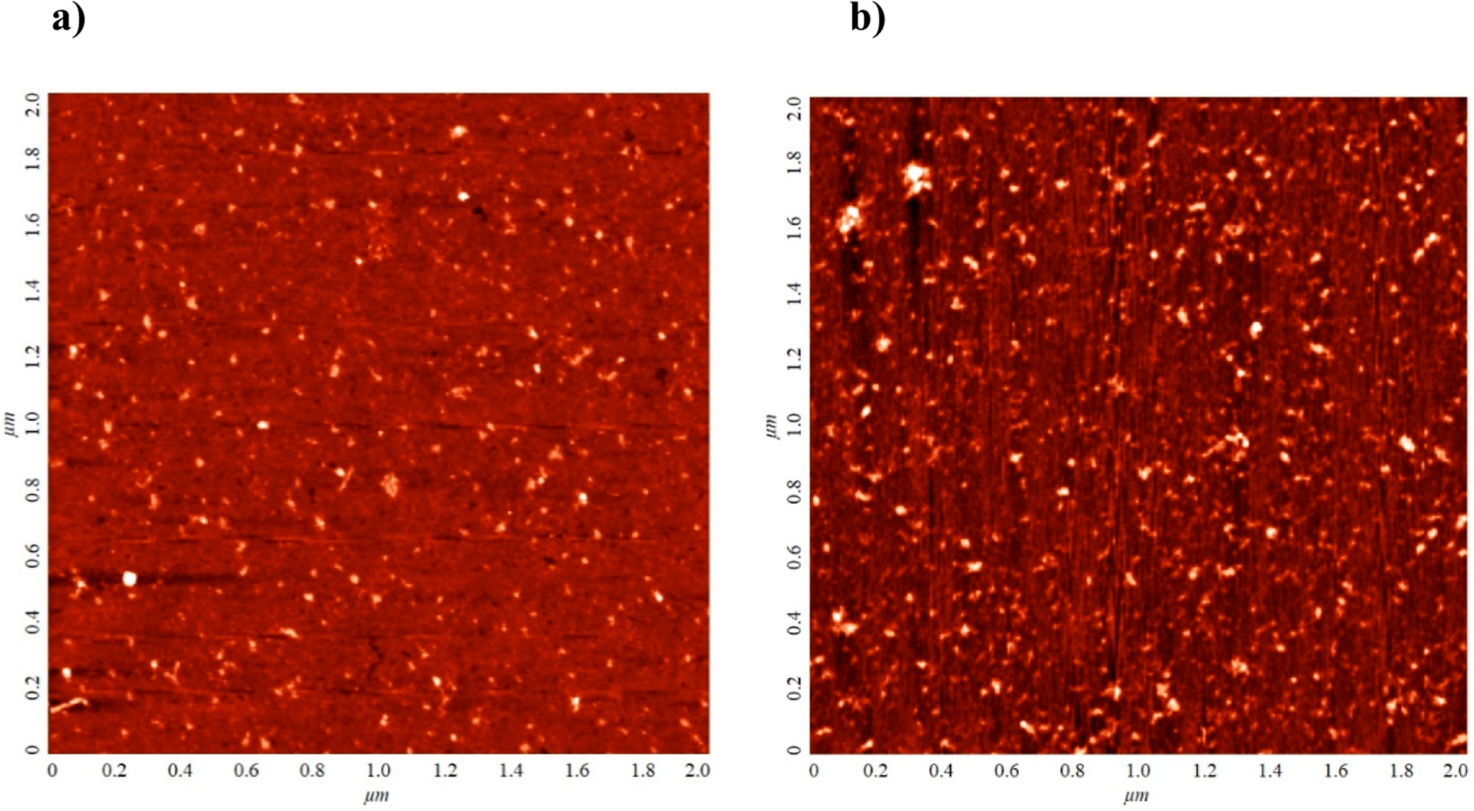
Vimentin layers at mica (AFM micrographs); adsorption conditions: pH = 3.5, *c*_b_ = 0.5 mg L^–1^, *t* = 15 min. (a) 0.001 M NaCl and (b) 0.01 M NaCl.

Vimentin adsorption kinetics at mica under diffusion transport was determined by the AFM method, similarly to previously published fibrinogen^30^ and human serum albumin^31^ data. Because of the molecularly smooth and homogeneous surface properties of mica, this method allows to determine the shape, 3-D topography, and surface concentration of molecules. In order to minimize protein aggregation during the adsorption, such investigations are performed at low protein concentrations, usually below 1 mg L^–1^. The experiments were performed at pH 3.5 and 1 and 10 mM NaCl, where the molecules are expected to bear a positive charge, given that their isoelectric point is equal to ca. 4.9.^32^ Typical AFM micrographs of vimentin aggregates at mica taken under these conditions are shown in Figure 1. One can observe that the average distance between vimentin aggregates is much larger than their dimensions, which enables us to determine their size and surface concentration.

**Figure 2:**
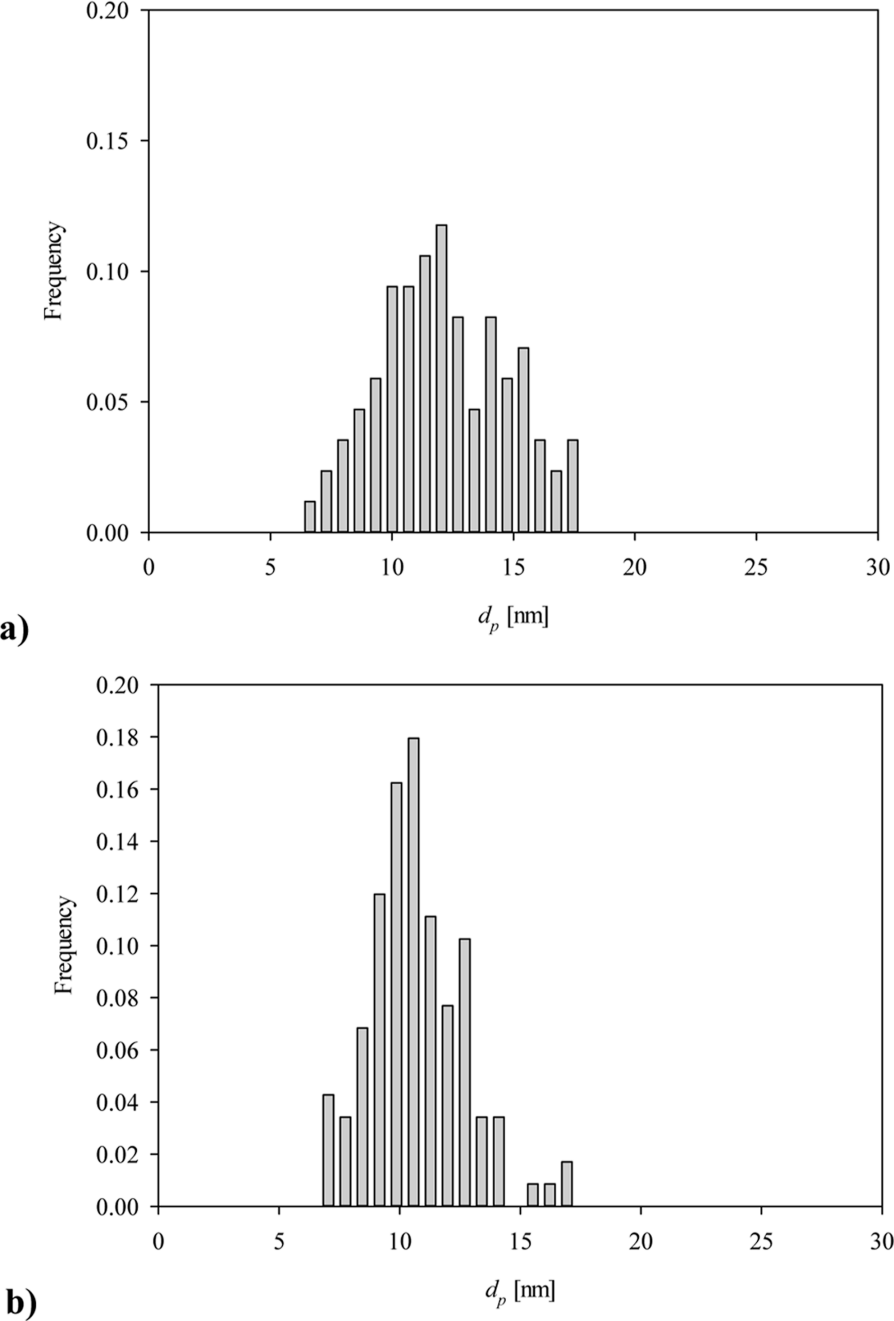
Histograms of vimentin aggregate size determined by AFM. (a) 0.001 M NaCl, average aggregate size 12 ± 2 nm, and (b) 0.01 M NaCl, average aggregate size 11 ± 1 nm.

A qualitative analysis of these micrographs indicates that the aggregates exhibit a regular quasi-spherical shape with a relatively small size spread. No elongated aggregates or filaments were observed in these micrographs. Quantitatively, the size distribution of aggregates was determined by measuring their dimensions in two perpendicular directions and taking an average value. The size histograms obtained in this way considering ca. 100 individual aggregates are shown in Figure 2. It was established that the average size of vimentin aggregates was 12 ± 2 and 11 ± 1 nm for 1 and 10 mM NaCl concentration, respectively. It is worth mentioning that these values are close to the diameter of intermediate filaments reported in the literature.^17−19^ It would be interesting to compare these aggregate sizes with that pertinent to the vimentin molecule monomer, which is, unfortunately, not available from experimental measurements. However, one can quite accurately estimate its size assuming that the monomer molar mass denoted by *M*_1_ is equal to 53.5 kg mol^–1^ (kDa).^15,24,33^ Thus, the volume of the monomer can be calculated from the dependence
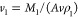
2where *Av* is the Avogadro number (6.02 × 10^23^) and ρ_1_ is the vimentin monomer density.

Taking the density value pertinent to globular proteins [e.g., human serum albumin (HSA)] equal to 1.36 g cm^–3^,^34^ one obtains from eq 2 that *v*_1_ = 65.3 nm^3^. The equivalent sphere diameter can be calculated as
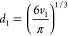
3

It is equal to 5.0 nm, which is an estimate of the real size of the vimentin monomer if its shape is approximated by a sphere.

Having the value of *d*_1_, it is possible to determine the characteristic cross-section of the monomer *S*_g_1__ = π*d*_1_^2^/4 = 20 nm^2^, and its diffusion coefficient of 9.8 × 10^–11^ m^2^ s^–1^ (see Table 1).

**Table 1:** Physicochemical Parameters of the Vimentin Molecule Monomer^a^

property, symbol unit	value	remarks
molar mass, monomer, *M*_1_, kg mol^–1^ [kDa]	53.5	from primary molecule structure^15^
density, ρ_p_, kg m^–3^	1.36 × 10^3^	estimated
specific volume, monomer, *v*_1_, nm^3^	65.3	calculated from molar mass and density, see footnotes
equivalent sphere (hydrodynamic) diameter, *d*_1_, nm	5.0	see footnotes
diffusion coefficient of the equivalent sphere (monomer), *D*_1_, m^2^ s^–1^	9.8 × 10^–11^	see footnotes, *T* = 298 K
geometrical cross-section of the equivalent sphere, *S*_g1_, nm^2^	20	see footnotes

a *v*_1_ = *M*_1_/ρ_p_*Av*; *d*_1_ = (6*v*_1_/π)^1/3^; *D*_1_ = *kT*/3πη*d*_1_; *S*_g_1__ = π*d*_1_^2^/4.

Comparing the monomer size with the average aggregate size determined from AFM, which is approximately 2 times larger, one may expect that they are composed of at least four vimentin monomers. Indeed, the dimensions of the tetramer forming a regular structure (see Table 2) are 10 × 10 nm with the diagonal equal to 14 nm, which results in an average diameter equal to 12 nm. This matches the experimental value within error bounds. On the other hand, for the hexagonal arrangement of four monomers, one obtains 10 × 13.7 nm as the aggregate dimensions. It should be mentioned that the presence of tetramers in vimentin solutions at low ionic strength was predicted and experimentally confirmed in previous investigations.^18,25^

**Table 2:**
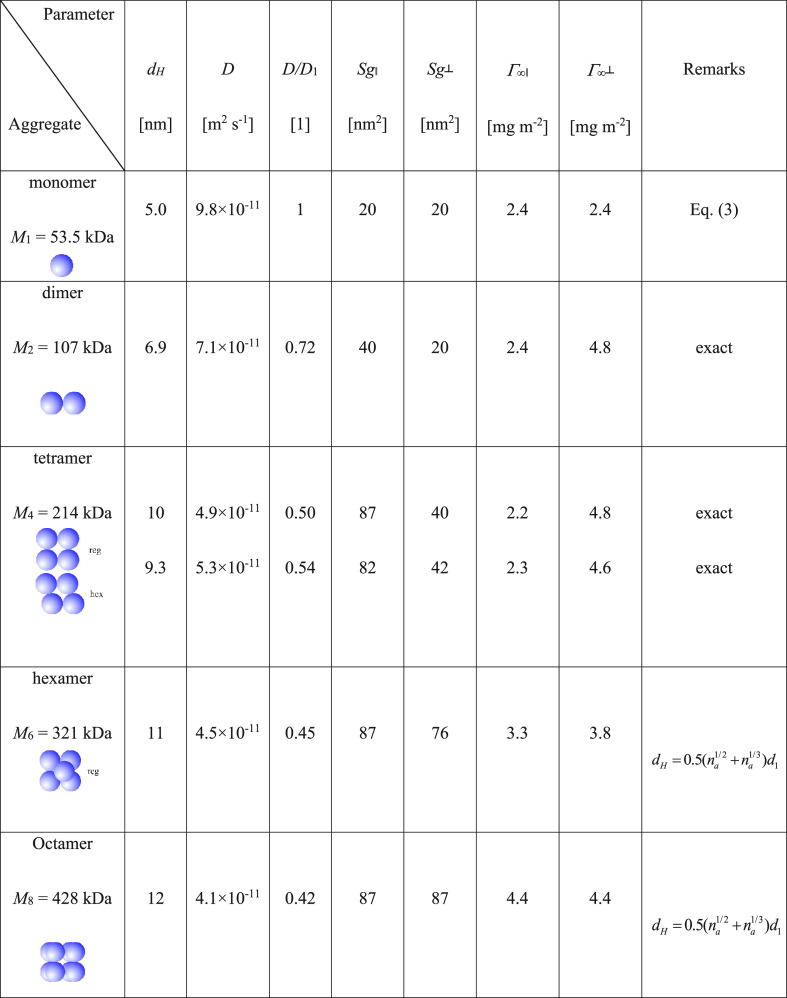
Physicochemical Characteristics of Vimentin Aggregates^a^

a *d*_H_—hydrodynamic diameter, *D*—diffusion coefficient, *S*_g∥_—cross-sectional area for the side-on adsorption, *S*_g⊥_—cross-sectional area for the end-on adsorption, Γ_∞∥_—maximum coverage for the side-on adsorption, Γ_∞⊥_—maximum coverage for the end-on adsorption, and *n*_a_—aggregation number

However, the analysis of aggregate sizes alone cannot unequivocally furnish the number of monomers they are composed of. This information can be acquired from measurements of protein adsorption kinetics performed in accordance with the protocol described in Materials and Methods using AFM under diffusion transport and QCM under convection-driven transport. The AFM measurements are especially useful because they directly yield the surface concentration expressed by *N*_a_ as a function of the adsorption time for various protein concentrations in the bulk and pHs. The surface concentration is connected with the aggregation number through the formula^35^
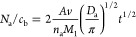
4where *n*_a_ is the aggregation number (thus, *M*_a_ = *n*_a_*M*_1_ is the aggregate molar mass), *D*_a_ is the aggregate diffusion coefficient, and *c*_b_ denotes the mass concentration of the vimentin solution.

The validity of eq 4 for a broad range of time was confirmed by performing numerical calculations of the exact transport equation with the blocking function stemming from the random sequential adsorption model (Supporting Information).

It is worth noticing that the rate of aggregate adsorption normalized to the monomer rate decreases as 1/*n*_a_(*D*_a_/*D*_1_)^1/2^ = 1/*n*_a_(*d*_H1_/*d*_Ha_)^1/2^, where *d*_Ha_ is the hydrodynamic diameter of the aggregate depending on their shape and *d*_H1_ is the hydrodynamic diameter of the monomer equal to 5.0 nm as calculated above. Values of hydrodynamic diameters for various aggregates numerically calculated are collected in Table 2. Thus, for the tetramer, one obtains 9.3 (hexagonal) to 10 (regular) nm, for the hexamer, 11 nm, and for the octamer 12 nm. As can be noticed, the hydrodynamic diameters only slightly depend on the aggregate size and shape. This indicates that the difference in the adsorption kinetics mainly stems from the aggregation number rather than from the change in the diffusion coefficient, which confirms an adequate precision of the kinetic method.

In Figure 4, results derived from these kinetic measurements are presented in the form predicted by eq 3, that is, as a function of *N*_a_/*c*_b_ on *t*^1/2^, and compared with theoretical results approximated for the monomer and for diverse aggregates (1 ≤ *n*_a_ ≤ 8). As one can see, the experimental results almost coincide with those theoretically predicted for the hexamer whose molar mass is equal to 321 kg mol^–1^. It is worth mentioning that this result harmonizes with that experimentally determined by Lopez et al.,^25^ where the molar mass of the starting solution of vimentin (determined by static light scattering) was equal to 330 kg mol^–1^. Moreover, it was shown that this value was fairly constant upon performing aggregation experiments induced by the addition of 10 mM KCl solution.

Obviously, the aggregation number predicted in the adsorption kinetic experiments should be treated as an average value because one can, for example, predict that an equal number of tetramers and octamers could yield the aggregation number equal to six. However, a spontaneous formation of vimentin aggregates indicates that the molecule charge and hydrophobicity are heterogeneously distributed. Thus, it is expected the inside part of aggregates is more hydrophobic and less charged compared to the outside part exposed to the solution, which is a usual phenomenon for ionic surfactants forming various micellar structures.^36^ If this assumption was valid, the formation of hexamers would be more probable, and as a result, their concentration would exceed that of the tetramer concentration. However, considering that our main goal was to determine the mechanism of vimentin monolayer formation, this interesting issue is not further elaborated in this work.

### Vimentin Adsorption Kinetics—QCM Measurements

Vimentin adsorption kinetic measurements were performed applying the quartz crystal microbalance (QCM) technique according to the protocol previously applied for fibrinogen^37^ and HSA.^38,39^ This method exhibits pronounced advantages making possible real time, in situ measurements of adsorption/desorption kinetics under various transport conditions (diffusion, flow). However, one should mention that the analysis of QCM measurements is a demanding task, because the oscillation frequency and the energy dissipation signals depend on the force exerted on the sensor rather than on the particle mass.^40,41^ If one considers a rigid contact, the net force includes the inertia component proportional to the particle mass and the hydrodynamic component, which can play a significant role. This effect is generally explained as the hydrodynamic solvent coupling leading to apparent hydration of protein layers.^37,39,40^

Typical QCM kinetic runs acquired for pH 3.5 and two ionic strengths of 10 and 150 mM NaCl are presented in Figure 4 as the dependence of the protein coverage expressed in mg m^–2^ (calculated using the Sauerbrey equation for the overtones 3, 5, 7, 9, and 11) on the adsorption time. It can be noticed that for 10 mM NaCl, the coverage rapidly increases, attaining a maximum value of 4.5 mg m^–2^ (for the third overtone) after 30 min of adsorption. During the desorption run, a minor decrease in the coverage was noticed, which attained the stationary value of 4 mg m^–2^. A similar behavior was registered for 150 mM NaCl (Figure 4b) with a slightly larger vimentin coverage after the desorption run equal to 5.5 mg m^–2^ (for the 3rd overtone). For the 11th overtone, the stationary coverages were equal to 3.2 and 5 mg m^–2^, respectively (for the 10 and 150 mM electrolyte concentrations).

It is worth mentioning that in the case of HSA (*M*_w_ 67 kg mol^–1^), the limiting QCM coverage equals 1.7 and 2.7 mg m^–2^ for 10 and 150 mM NaCl concentrations, respectively.^38^ Considering the solvent coupling effect, this corresponded to the dry coverage equal to 0.7 and 1.4 mg m^–2^ for 10 and 150 mM NaCl concentrations, respectively. Using the value of the hydration function determined in ref (38) for albumin, one can predict that the dry coverage of vimentin (calculated as an average from the 3rd and 11th overtones) is equal to 1.8 and 2.6 mg m^–2^ for 10 and 150 mM NaCl, respectively. As can be noticed, these values fairly well agree with those theoretically predicted for the side-on adsorption of the tetramer (see Table 2). It should also be mentioned that the increase in the protein coverage with the electrolyte concentration can be attributed to the decreased range of the repulsive electrostatic interactions among adsorbed molecules.^38,42,43^ The side-on adsorption mechanism was further confirmed by the topographical analysis of the vimentin layers adsorbed on the silica sensors in the QCM cell (see the inset in Figure 4), quantitatively characterized on the basis of the rms factor (Supporting Information). It was shown that the average thickness of the aggregates was equal to 5.1 nm, which corresponds to the predicted tetramer thickness.

Analogous vimentin adsorption/desorption kinetic experiments were performed at pH 7.4, adjusted with the PBS buffer at ionic strengths equal to 10 and 150 mM. The results shown in Figure 5a,b exhibit similar features as previously observed at pH 3.5; that is, initially, the coverage rapidly increases, attaining a maximum value of 9 mg m^–2^ (for the third overtone) after 30 min of adsorption. During the desorption run, the coverage decreases to 8.5 mg m^–2^. For the 11th overtone, the stationary coverages were equal to 6.5 mg m^–2^, respectively. It is interesting to mention that in contrast to pH 3.5, the differences in the stationary coverages between 10 and 150 mM ionic strength were much smaller, within experimental error bounds. Considering the solvent coupling effect as previously mentioned, one can predict that the dry coverage of vimentin (calculated as an average from 3rd and 11th overtones) at pH 7.4 is equal to 3.7 mg m^–2^ for ionic strength in the range of 10 to 150 mM. As can be noticed, this maximum coverage is larger than that determined at pH 3.5 and agrees with theoretically predicted for the end-on adsorption of the hexamer (see Table 2).

**Figure 3:**
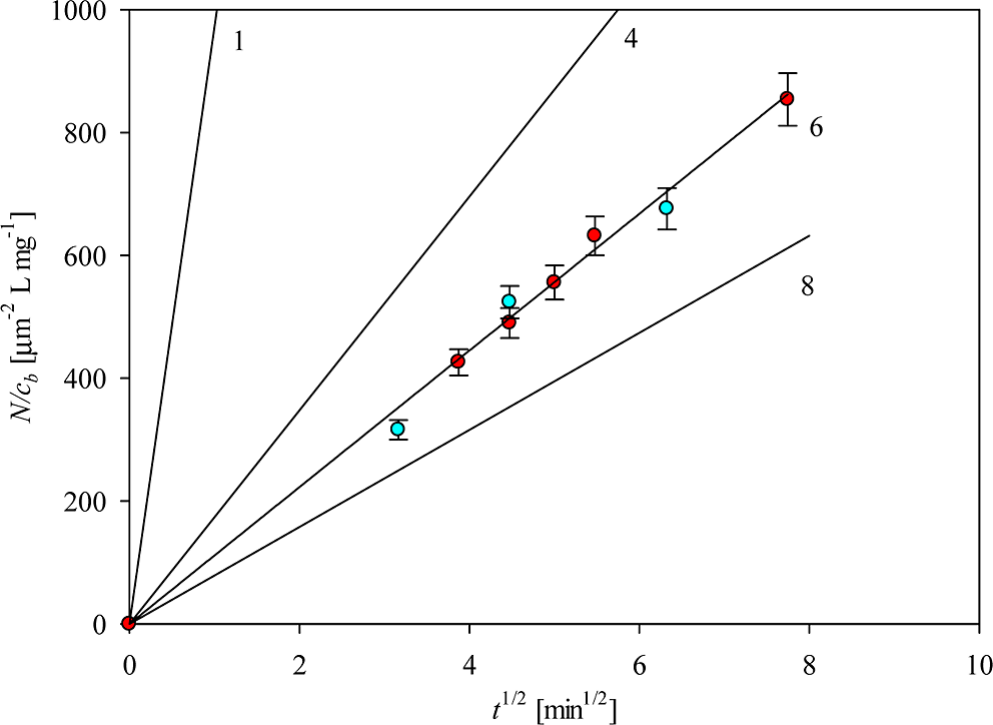
Normalized surface concentration of vimentin vs the square root of the time of adsorption *t*^1/2^. The points express experimental results achieved from AFM (adsorption conditions pH = 3.5 (● red), and 7.4 (● blue), 10 mM NaCl, bulk protein concentration *c*_*b*_ = 0.5–1 mg L^–1^. The black lines display the theoretical results obtained by numerical solution of the diffusion transport equation for 1—the monomer, 4—the tetramer, 6—the hexamer, and 8—the compact octamer.

**Figure 4:**
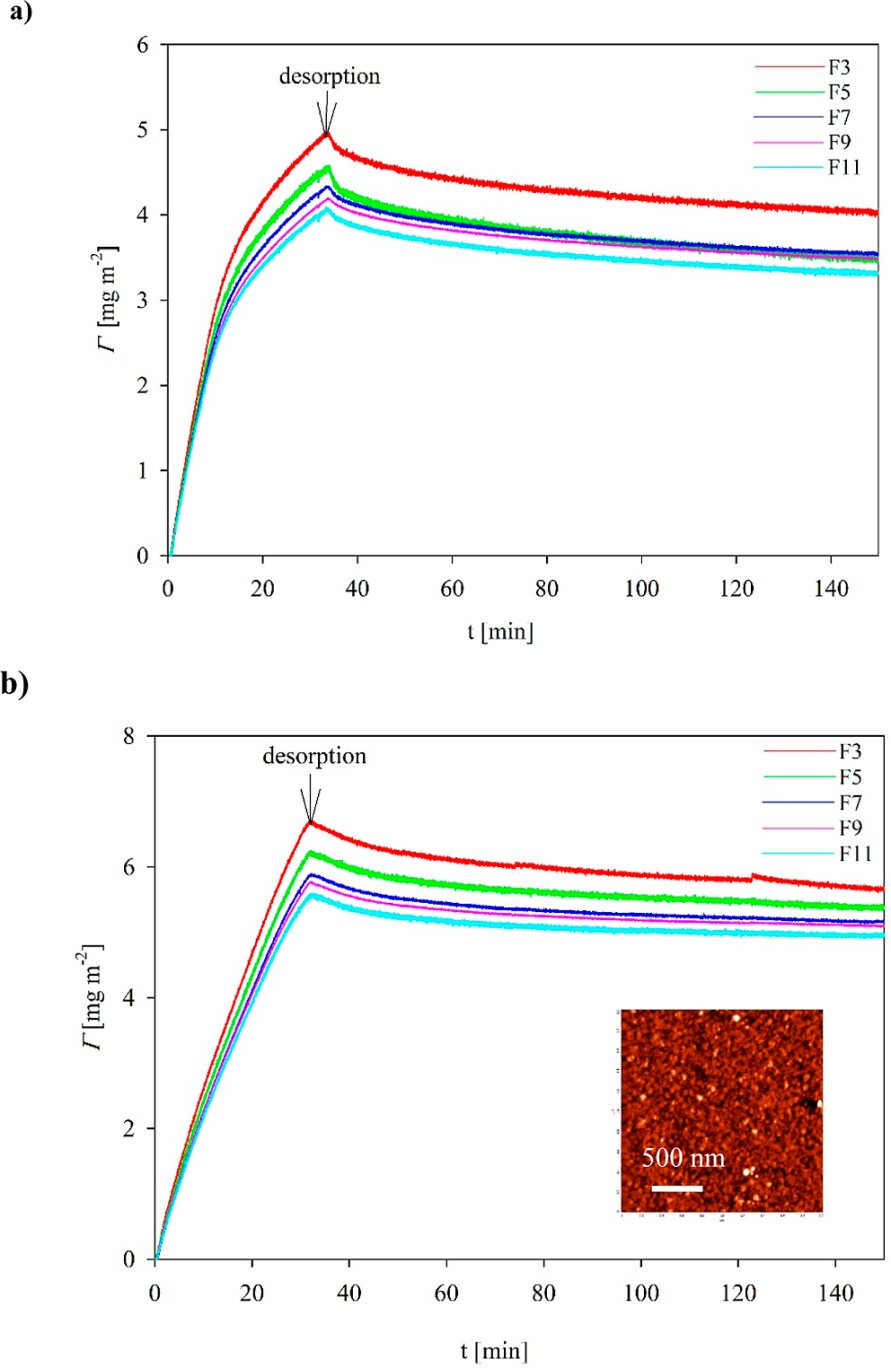
Kinetics of vimentin adsorption/desorption presented as the dependence of the coverage (calculated for the overtones 3, 5, 7, 9, and 11) on the time; adsorption conditions pH 3.5, bulk protein concentration 5 mg L^–1^, flow rate 8.3 × 10^–4^ cm^3^ s^–1^; (a) NaCl electrolyte concentration 10 mM, (b) NaCl electrolyte concentration 150 mM. The inset shows AFM micrographs of protein adsorbed at the QCM sensor after the adsorption run.

**Figure 5:**
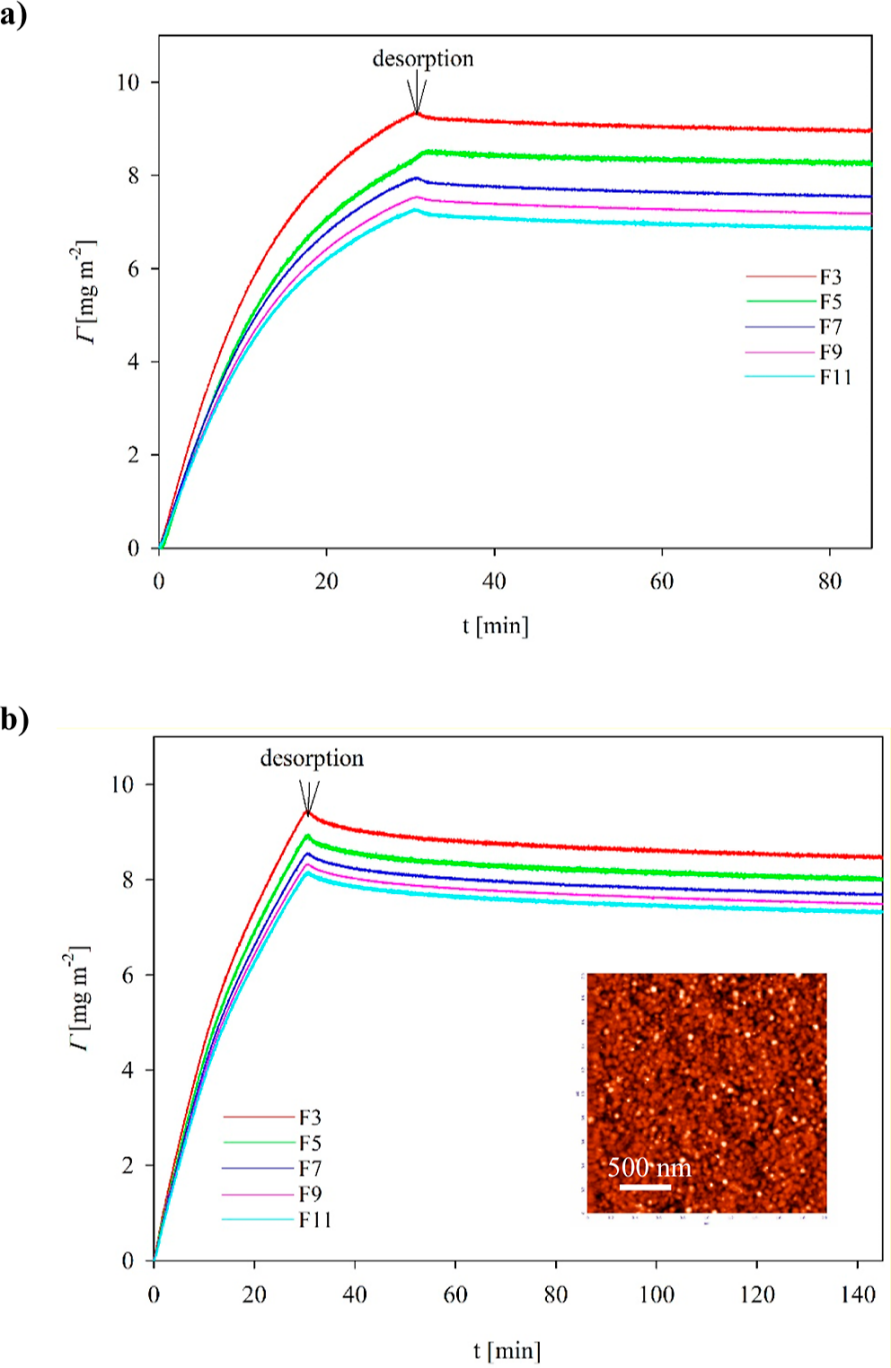
Kinetics of vimentin adsorption/desorption expressed as the dependence of the coverage (calculated using the Sauerbrey equation for various overtones) on the deposition time; adsorption conditions: pH 7.4, bulk protein concentration 5 mg L^–1^, flow rate: 8.3 × 10^–4^ cm^3^ s^–1^. (a) pH 7.4, *I* = 10 mM, (b) pH 7.4 (PBS), *I* = 150 mM. The inset shows AFM micrographs of vimentin layers at the silica sensor after the adsorption run.

The AFM and the in situ QCM kinetic results shown in Figures 2–5 confirm efficient adsorption of vimentin on the negatively charged surfaces because the zeta potential of mica and silica at pH 7.4 and 10 mM ionic strength was equal to −70 and −45 mV, respectively. This indicates that there exist positive charge patches at vimentin aggregates at pH 7.4, whereas the average aggregate charge is negative. Moreover, the end-on mechanism of the adsorption suggests that the positive charge is placed at the perimeter of the vimentin aggregates, whereas the inside part is negatively charged. One can hypothesize that such heterogeneous charge distribution can be responsible for the aggregation of vimentin, leading to various fibril-like structures observed in previous works.^12−15,18,19^

It should also be mentioned that such a heterogeneous charge distribution was confirmed for the fibrinogen molecule, performing theoretical modeling, solution viscosity,^44^ streaming potential,^30^ and adsorption kinetics measurements.^45^

More information about the charge distribution over vimentin aggregates can be derived by applying electrokinetic methods, primarily the LDV involving polymer carrier particles of well-defined and homogeneous charge distribution. The results of these experiments are discussed in the next section.

### Vimentin Corona Formation at Polymer Particles

Adsorption of vimentin at negatively charged S800 particles, referred later to as corona formation in accordance with commonly used nomenclature,^46,47^ was performed according to the following procedure. Equivalent volumes of the vimentin solution with a concentration ranging from 0.2 to 2 mg L^–1^ and the S800 particle suspensions of the concentration equal to 50 or 100 mg L^–1^ were mixed over 15 min. In separate experiments, it was confirmed that such mixing time was adequate for full corona formation, in accordance with the theoretical prediction of the relaxation time of this process given in the Supporting Information. After finishing an adsorption run, the electrophoretic mobility of the microparticles with the corona was measured by the LDV and the corresponding zeta potential was calculated using the Smoluchowski equation. Primarily in these experiments, the dependence of the zeta potential of the particles on the vimentin bulk concentration was determined. In order to facilitate their quantitative interpretation, the nominal corona coverage Γ was calculated using the mass balance equation^47−49^
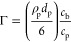
5where ρ_p_ is the polymer particle density, *d*_p_ is the particle diameter, and *c*_p_ is the particle concentration in the bulk.

**Figure 6:**
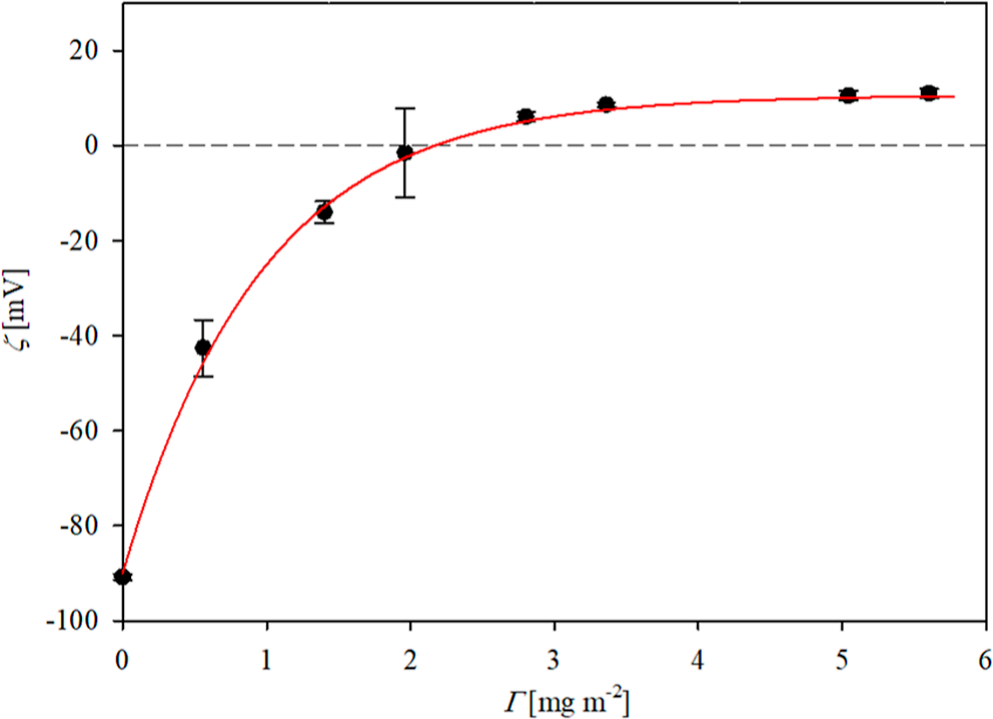
Dependence of the zeta potential of vimentin at negatively charged polymer particles (S800) on the nominal protein coverage calculated from eq 5; adsorption conditions: pH 3.5 and 10 mM NaCl. The points denote experimental results obtained from the LDV measurements, and the red line shows the theoretical results predicted from the electrokinetic model using eq 6.

In Figure 6, the dependence of the zeta potential of the vimentin corona on the nominal coverage calculated from eq 5 obtained at pH 3.5 and in a 10 mM electrolyte is shown. It can be observed that the initially negative zeta potential rapidly increases with the vimentin coverage and becomes positive for a Γ larger than 2 mg m^–2^. However, for still larger coverages, the change in the zeta potential becomes rather minor, and finally, a plateau value equal to 10 mV is attained. The primary experimental data presented in Figure 6 were theoretically analyzed using the electrokinetic model formulated in ref (50). This enabled us to formulate the following formula for the zeta potential of the protein corona at polymer particles ζ_*c*_(Θ)
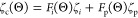
6where Θ is the absolute (dimensionless) coverage of protein molecules calculated from the formula
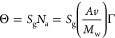
7*S*_g_ is the characteristic cross-sectional area of the vimentin molecule aggregate (see Table 2), ζ_*i*_ is the zeta potential of carrier particles, ζ_*p*_ is the bulk protein zeta potential, and *F*_*i*_(Θ) and *F*_p_(Θ) are the dimensionless functions. The *F*_*i*_ function describes the damping of the flow near the particle surface by the adsorbed molecule layer, and the *F*_p_ function characterizes the contribution to the zeta potential stemming from the molecules.^50^

The results obtained from eqs 6 and 7 for the hexamer using the molar mass and geometrical cross-section areas given in Table 2 are presented as a red line in Figure 6. As one can observe, they appropriately reflect the experimental run, indicating that the maximum coverage is equal to 3.0 mg m^–2^, which agrees with the theoretical value predicted for the hexamer adsorbing side-on. To increase the precision of the maximum coverage determination, a concentration depletion method exploiting AFM was used.^51^ This procedure gives the remaining concentration of vimentin in the solution after the adsorption on the particles. Knowing the initial and the residual concentration, the true coverage of adsorbed vimentin can be calculated from eq 5. Thus, we obtained 2.6 mg m^–2^, which is smaller than the above value probably due to the desorption of vimentin molecules during the application of the concentration depletion method, analogously as observed in the QCM runs.

**Figure 7:**
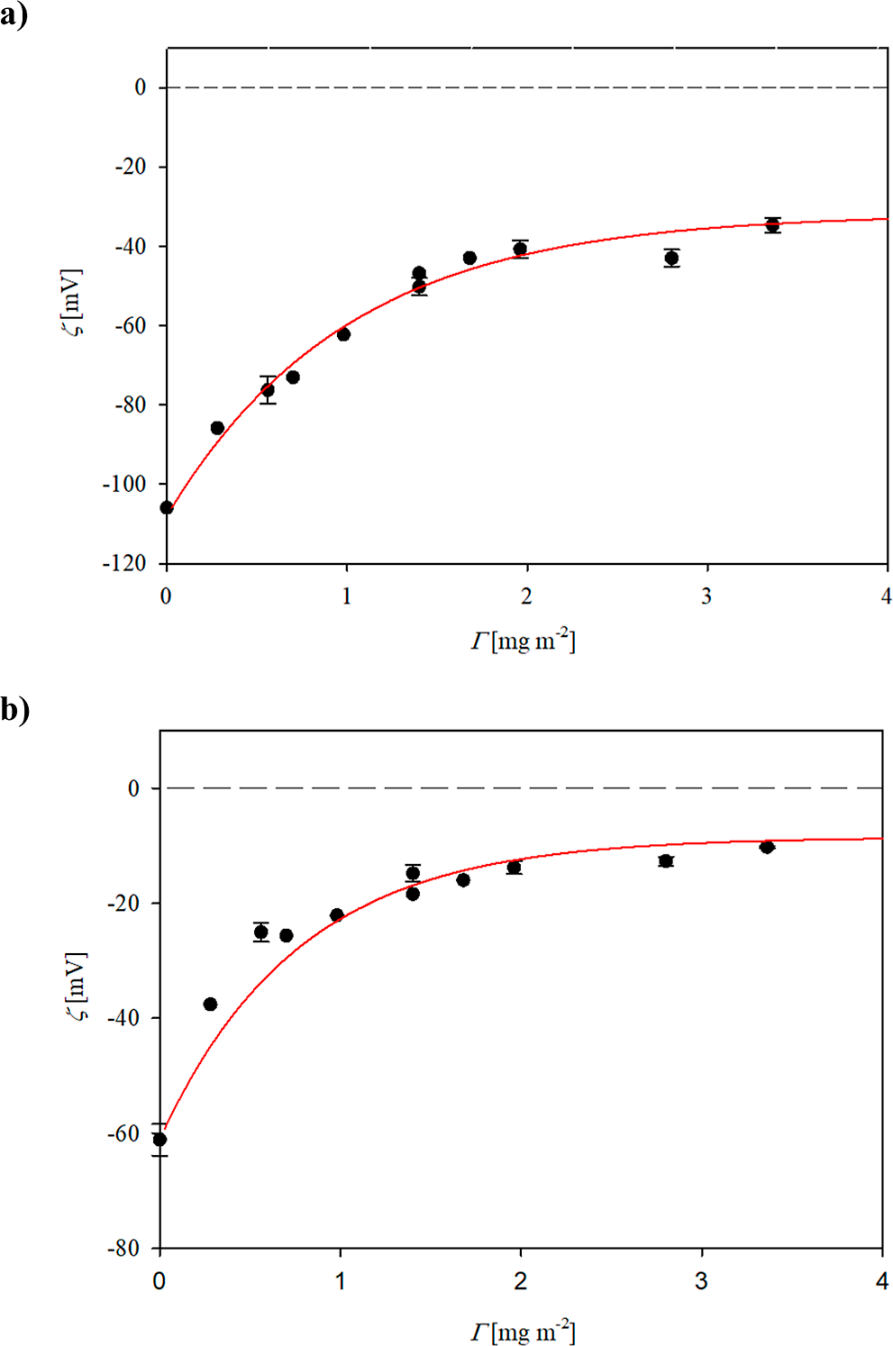
Dependencies of the zeta potential of vimentin at negatively charged polymer particles (S800) on the nominal protein coverage calculated from eq 5; adsorption conditions: pH 7.4 (PBS); (a) 10 mM ionic strength and (b) 150 mM ionic strength. The black points denote experimental results obtained from the LDV measurements, and the red lines show the theoretical results calculated from the electrokinetic model using eq 6.

Analogous measurements of vimentin corona formation at pH 7.4 are plotted in Figure 7. One can observe that the initially negative zeta potential of particles rapidly increases with the coverage and stabilizes at the value equal to −35 and −12 mV for 10 and 150 mM electrolyte concentrations, respectively. As before, the experimental results presented in Figure 7 are adequately interpreted using the electrokinetic model, where the zeta potential of the vimentin corona is calculated from eqs 6 and 7 for the hexamer and for the 10 and 150 mM electrolyte concentration, respectively. The maximum coverage equals ca. 3.5 mg m^–2^, which is in agreement with the theoretical value predicted for the hexamer adsorbing side-on and with the previous value obtained from QCM measurements.

**Figure 8:**
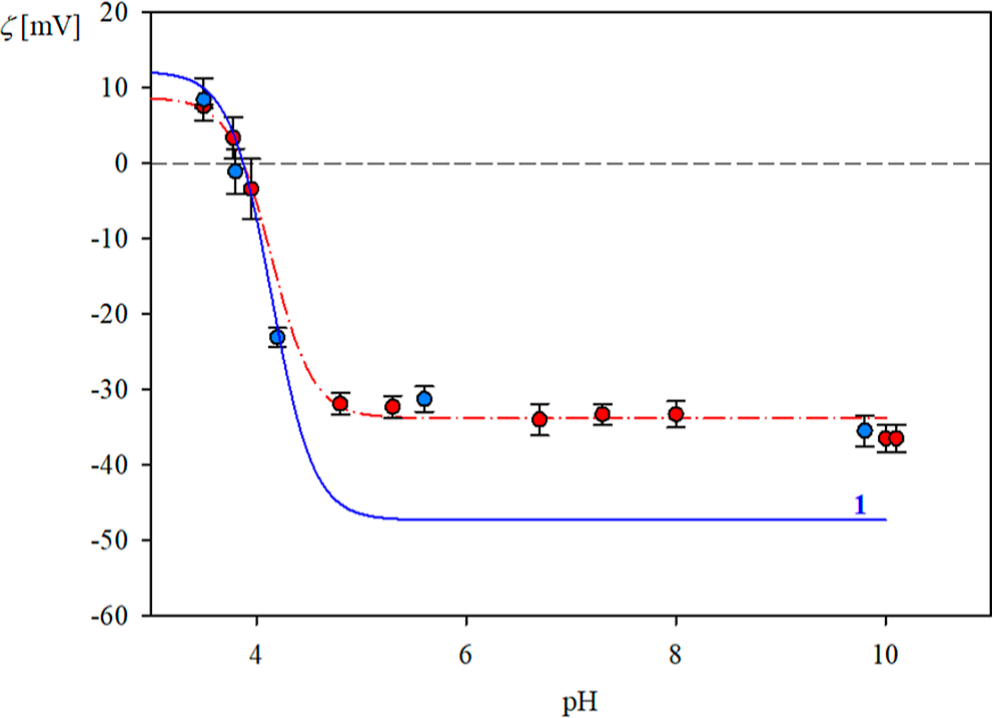
Dependence of the zeta potential of S800 particles with vimentin corona on the coverage equal to 3 mg m^–2^ on pH; the points show experimental data obtained using the LDV method for 10 mM NaCl, (● red)—pH increase; (● blue)—pH decrease, interpolated by the dashed line. The solid (1) line represents the bulk aggregate zeta potential calculated from eq 8.

It was also determined that the particle suspensions with vimentin coronas were stable longer than 24 h which facilitated determining their electrophoretic mobility and, in consequence, their zeta potential as a function of pH using the Smoluchowski equation. The results of such measurements, performed for the corona adsorbed at pH 3.5 with the coverage of 3 mg m^–2^, are shown in Figure 8. As one can notice, the zeta potential of the particles becomes negative at a pH larger than 4, and then it attains the value of −37 mV, which remains stable for a pH up to 10. If the zeta potential of the particles with the vimentin corona is known, one can calculate the bulk zeta potential of vimentin aggregates by rearranging eq 6 to the resulting form
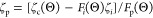
8

Considering that for the large corona coverage, the *F*_*i*_(Θ) function vanishes and the *F*_p_(Θ) function reaches 0.71,^51^ one can predict that ζ_p_ = 1.4ζ(Θ).

The results calculated from eq 8 and shown as a blue line in Figure 8 indicate that the zeta potential of aggregates varies between 15 mV at pH 3.5 to −52 mV at pH 7.4 (PBS). One should mention that the determination of the vimentin aggregate zeta potential is impractical using LDV because this would require protein concentration in the bulk of about 500 mg L^–l^, resulting in instability of the solution.

The zeta potential determined in this way can also be used to establish the net electrokinetic charge of aggregates as a function of pH from the following dependence^52^
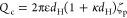
9where ε is the permittivity of the electrolyte and κ^–1^ is the thickness of the electric double layer.

Using eq 9 the number of elementary charges per single aggregate can be calculated. It was equal to 22 and −63 for pH 3.5 and 7.4, respectively, which corresponds to the surface charge of 0.17 and −0.7 e nm^–2^, respectively. The latter value is similar to that reported in ref (53) for vimentin fibrils. It is to remember, however, that at pH 7.4, a significant amount of positive charge is predicted from the above deposition kinetic measurements, although, at present, it cannot be quantified.

The results obtained in this work confirm that it is feasible to fabricate stable particle suspensions with a vimentin corona of a desired coverage and zeta potential only using 0.1 nM quantities of the protein. Such layers can be exploited for investigating interactions with various macromolecule ligands.

## Conclusions

The AFM investigations of vimentin adsorption at mica provided reliable data about its basic physicochemical properties. It was established that vimentin in solution at pH 3.5 and 7.4 mainly consists of aggregates in the form of compact tetramers and hexamers having a size equal to 11–12 nm that agrees with the cross-sectional diameter of intermediate vimentin filaments reported in the literature.

These results were confirmed by vimentin adsorption measurements at planar surfaces carried out using QCM and at polymer particles, leading to corona formation studied by the LDV method. In both cases, it was established that the aggregates efficiently adsorb at negatively charged silica sensors and polymer particles, both at pH 3.5 and 7.4, forming compact layers of maximum coverage up to 3.5 mg m^–2^. One can, therefore, argue that the charge distribution over vimentin aggregates at pH 7.4 is heterogeneous, with positive charge patches located at the perimeter of the aggregates, whereas the inside part remains negatively charged. It is hypothesized that such heterogeneous charge distribution can promote the formation of various fibril-like vimentin structures observed in previous works.^17−26^

It was also shown that a quantitative interpretation of vimentin corona formation at polymer particles is feasible in terms of the electrokinetic model. The dependence of the corona zeta potential on the coverage acquired in this way can be exploited to develop a robust procedure for preparing functionalized particles with controlled corona density. The particles can be used for investigations of vimentin molecule interactions with various ligands comprising immunoglobulins, bacteria virulence factors, and spike proteins of viruses.

## Supporting Information Available

Characteristics of particles and substrates, adsorption kinetics investigations, AFM measurements, QCM measurements, theoretical analysis of adsorption kinetics, and vimentin corona formation at polymer particles (PDF)
