# Supplementary material for: Human Vimentin Layers on Solid Substrates: Adsorption Kinetics and Corona Formation Investigations
Source: Biomacromolecules. 2022 Jul 13;23(8):3308–17. doi: 10.1021/acs.biomac.2c00415 (PMC9364323; doi:10.1021/acs.biomac.2c00415)
Supplement: Supplementary file 1 — bm2c00415_si_001.pdf [file bm2c00415_si_001.pdf]

Supporting Information

Human Vimentin Layers at Solid Substrates:

Adsorption Kinetics and Corona Formation Investigations

Monika Wasilewska[1*], Paulina Żeliszewska[1], Katarzyna Pogoda[2], Piotr Deptuła[3], Robert Bucki[3*], Zbigniew Adamczyk[1*]

[1]*J. Haber Institute of Catalysis and Surface Chemistry Polish Academy of Science, Niezapominajek 8, 30-239 Cracow, Poland*

[2]*Institute of Nuclear Physics, Polish Academy of Sciences, PL-31342 Kraków, Poland*

[3]*Department of Medical Microbiology and Nanobiomedical Engineering, Medical University of Białystok, PL-15222 Białystok, Poland*

* Co-Corresponding authors

Contents:

1.Characteristics of particles and substrates

2. Adsorption kinetics investigations

    2.1 AFM measurements

    2.2 QCM measurements

3. Theoretical Analysis of Adsorption Kinetics

4. Vimentin Corona Formation at Polymer Particles

1. Characteristics of particles and substrates

Bulk characteristics of the polystyrene particles (hereafter referred to as PS) used as carriers in the vimentin corona formation experiments were performed using the LDV (electrophoretic mobility) and the DLS (diffusion coefficient) measurements. Primarily, the particle electrophoretic mobility μ_e was measured for different ionic strengths. These data were converted to the zeta potential vs pH dependencies and are presented in Fig. S1a. As can be seen, the zeta potential of polymer particles at pH 3.5 was equal to -100 ± 5 and -59± 5 for the NaCl concentration of 10 and 150 mM, respectively. At pH 7.4 (PBS) the zeta potential was equal to -90 ± 10 and -65 ± 5 mV for the NaCl concentration of 10 and 150 mM, respectively. On the other hand, the particle size was determined from the Stokes-Einstein formula exploiting the diffusion coefficient values acquired by DLS measurements. It was established that the hydrodynamic diameter of the PS particles at pH range 3.5 – 9 was equal to 830 ± 20 and 820 ± 20 nm for 10 and 150 mM NaCl, respectively.

The zeta potential of mica sheets and silicon/silica plates used in vimentin adsorption kinetics measurements was determined by streaming potential measurements in a four-electrode microfluidic cell according to the procedure described in Refs. [1, 2]. Initially, the streaming potential was measured using a pair of reversible electrodes as a function of the hydrostatic pressure difference ΔP. Subsequently, the streaming potential was converted to the zeta potential ζ using the Smoluchowski relationship [3] considering the correction for the surface conductivity. The dependence of the zeta potential on pH is graphically shown in Fig. S1b. One can see that for bare mica sheets, the zeta potential decreases from -45 mV at pH 3.5 to -70 mV at pH 7.4 (10 mM NaCl). Analogously for silicon/ silica the zeta potential was equal to -10 and -45 mV at pH 3.5 and 7.4 (10 mM NaCl).

Additionally, thorough topographic characteristics of mica sheets and silica sensors were carried out using ambient air AFM imaging. It was confirmed that the root mean square (rms) of mica sheets was equal to 0.1 nm, whereas for the QCM sensors it was equal to 0.8 nm.

Part a)

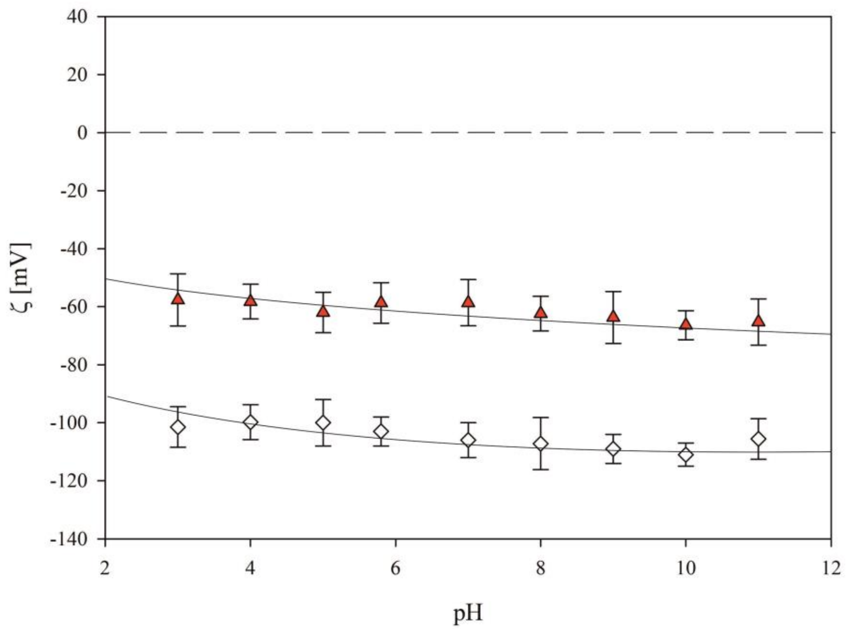

Part b)

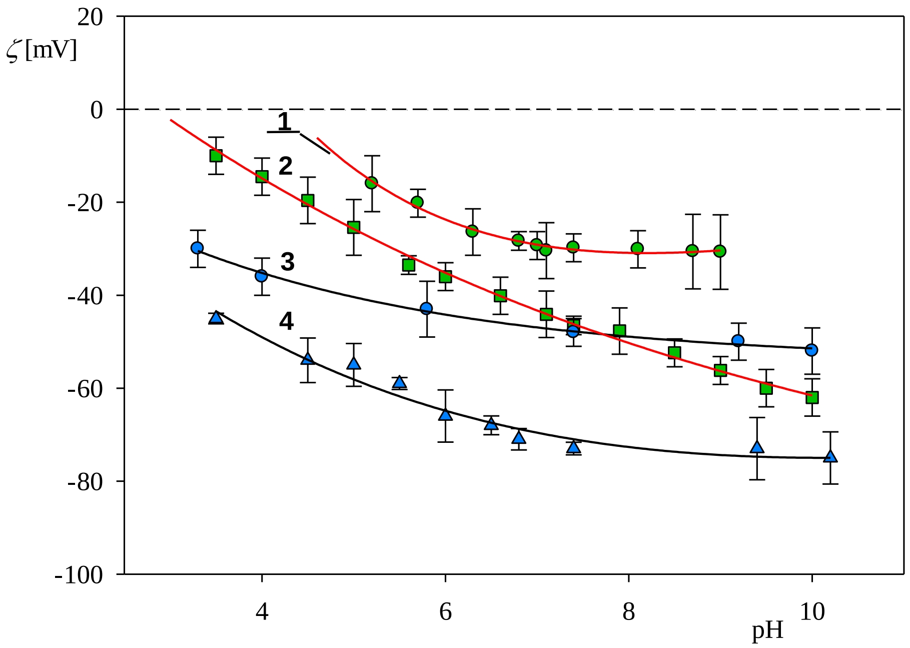

Fig. S1. Part a. The dependence of the zeta potential of PS particles on pH determined by the LDV method. The solid lines represent fits of experimental data.

1. 150 mM NaCl (▲)
2. 10 mM NaCl (◊)

Part b. The dependence of the zeta potential of mica and silica on pH determined by the streaming potential method,

1. bare silica, 150 mM NaCl [2]
2. bare silica, 10 mM NaCl
3. bare mica, 150 mM NaCl [1]
4. bare mica, 10 mM NaCl

The solid lines represent fits of experimental data.

2. Adsorption Kinetics Investigations

2.1. AFM measurements

Adsorption kinetics of vimentin at mica sheets under diffusion transport was determined by the AFM method, according to the procedure previously applied for fibrinogen [4] and human serum albumin [5]. Because of molecularly smooth and exceptionally homogeneous surface properties of mica, this methods exhibits pronounced advantages, enabling to determine the dimensions, shape and the number of protein molecules per unit area of the interface (the surface concentration of molecules). In these investigations diluted protein solutions were used, usually below 1 mg L^{-1}, that minimized aggregation during the adsorption run. Measurements were carried out at pH 3.5 and 7.4 for different NaCl concentrations. Typical AFM micrographs of vimentin aggregates at mica taken under these conditions are shown in Fig. S2. One can observe that the aggregates are separated from each other by an average distance much larger than their dimensions, which enables to determine their size and surface concentration.

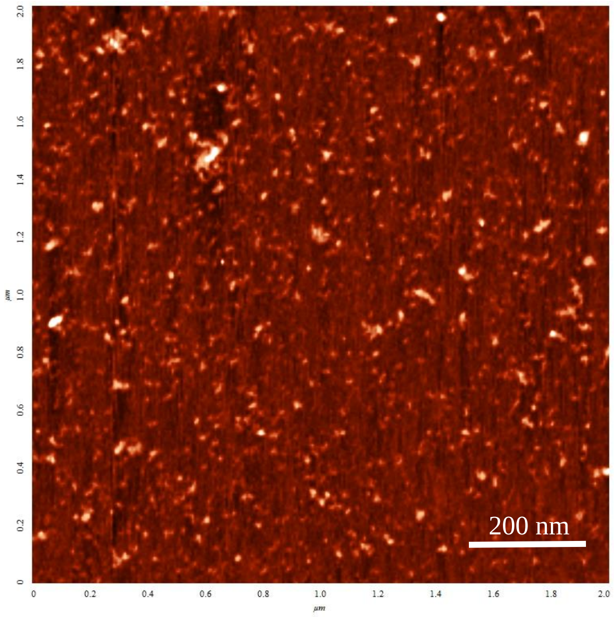

Fig. S2. Vimentin aggregates at mica, AFM micrographs; adsorption conditions: pH 3.5, c_b = 0.5 g L^{-1}, diffusion adsorption time t = 15 min, 10 mM NaCl.

A qualitative analysis of these micrographs proved that the aggregates exhibited a regular quasi-spherical shape with a relatively small size spread. No elongated aggregates or filaments were observed. Quantitatively, the size distribution of aggregates was determined by measuring their dimensions in two perpendicular directions and taking an average value. The size histograms obtained in this way considering ca. 100 individual aggregates allowed to determine their average size, which was equal to 12 ± 2 and 11±1 nm for 10 and 150 mM NaCl concentration, respectively.

Additionally, using AFM, the surface concentration of aggregates, denoted by N_a, was determined as a function of the adsorption time for various bulk protein concentrations and pHs. The experiments were carried out under diffusion-controlled transport under thermostated conditions. The kinetics runs acquired in this way were used to determine the average vimentin molecule aggregation number using the formula derived in the third section.

2.2. QCM Measurements

Vimentin adsorption kinetic measurements were also performed using QCM according to the procedure previously applied for other globular proteins [6]. This method exhibits pronounced advantages enabling real-time, *in situ* measurements of adsorption/desorption kinetics under different transport conditions (diffusion, flow) with unprecedented precision [7].

Typical QCM kinetic runs acquired for pH 3.5 and two different ionic strengths of 10 and 150 mM NaCl are shown in Fig. S3 as the dependence of the normalized frequency for the overtones 3, 5, 7, 9, 11 on the adsorption time

Using the primary $\Delta f / n_0$ data, the QCM coverage of aggregates was calculated from the Sauerbrey equation [7]

$$\Gamma_Q = C_Q(-\Delta f / n_0) \tag{S1}$$

where $C_Q = \frac{Z_q}{2 f_F^2}$ is the Sauerbrey constant equal to 0.177 (mg m^{-2} Hz^{-1}) for the fundamental frequency f_F = 5×10^6 Hz and Z_q is the acoustic impedance of the quartz sensor equal to 8.8×10^6 kg m^{-2} s^{-1}.

a)

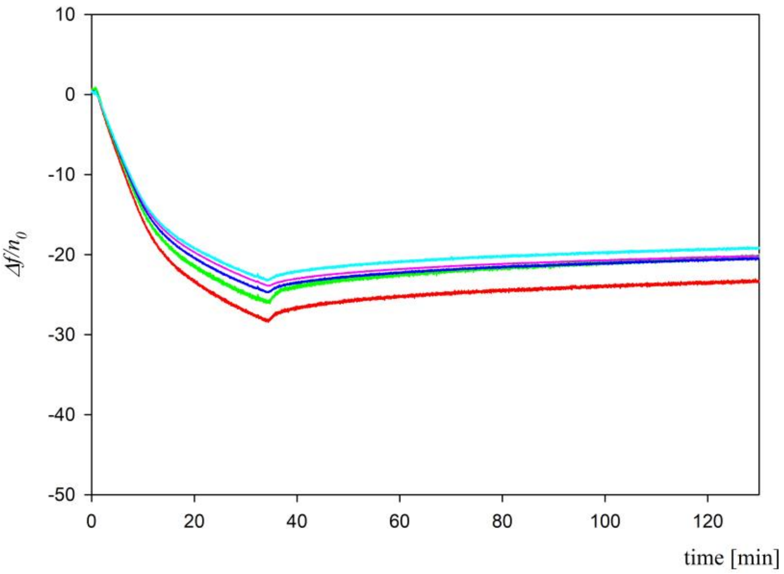

b)

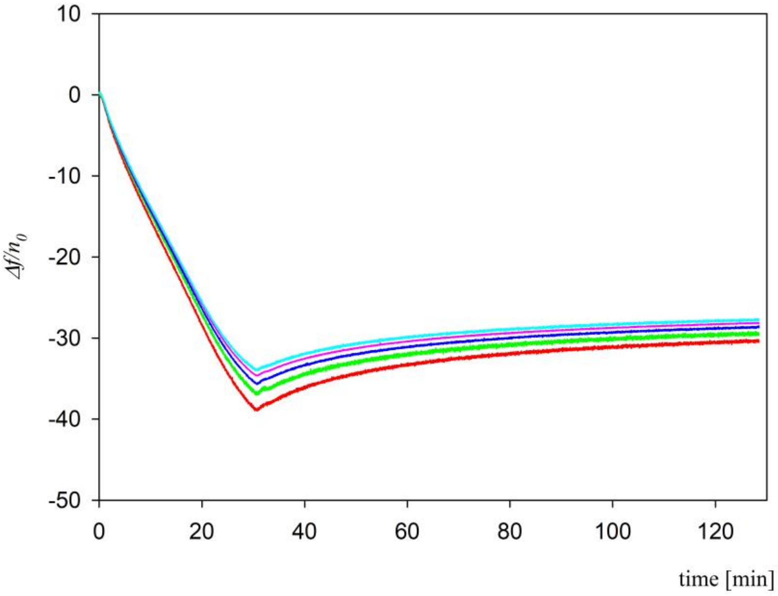

Fig. S3. Kinetics of vimentin adsorption /desorption at the silica sensor expressed as the dependence of the normalized frequency shift $\Delta f\,/\,n_0$ for the overtones 3 – red, 5 – green,7 – blue,9 – pink,11 – cyan)

on the time; adsorption conditions pH 3.5, bulk protein concentration 5 mg L^{-1}, flow rate 8.3×10^{-4} cm^3 s^{-1}; Part a) 10 mM NaCl, part b) 150 mM NaCl.

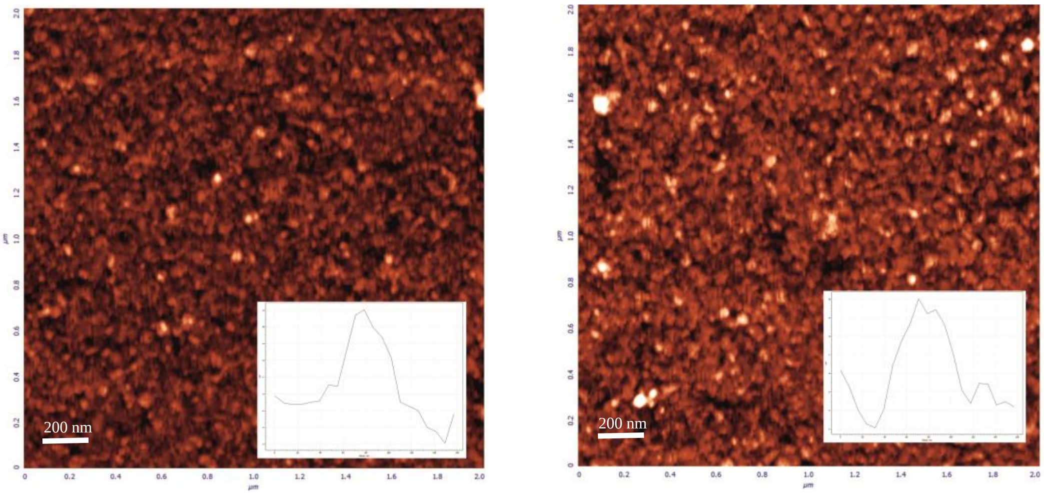

Fig. S4. AFM micrographs of vimentin layers at silica sensor, adsorption conditions as in Fig. S3. Part a, 10 mM NaCl, Part b, 150 mM NaCl. The insets show a fragment of the height profiles through the protein layer.

The topography of vimentin layers on the silica sensor imaged by AFM after completing the QCM measurements are presented in Fig. S4. The analysis of height profiles indicates that the vertical dimension of adsorbed aggregate is ca. 5 nm.

A more precise characterization of vimentin layers was performed by calculating the root mean square roughness (*rms)* defined as

$$rms^2 = \frac{1}{S}\int_s \left(h(\mathbf{r}_s) - \bar{h}\right)^2 d\mathbf{r}_s = \frac{1}{S}\int_s h^2(\mathbf{r}_s) - \bar{h}^2 \qquad \text{(S2)}$$

where S is the geometrical area of the AFM image, $h(\mathbf{r}_s)$ is the local height of the surface profile measured relatively to the reference value h_0, $\mathbf{r}_s$ is the surface position vector, $\bar{h}$ is the average height of the protein layer defined as

$$\bar{h} = \frac{1}{S}\int_s h(\mathbf{r}_s)\, d\mathbf{r}_s \tag{S3}$$

It is shown in Ref [8] that for convex particles the surface *rms* is given by the formula

$$rms = \left[c_1\Theta\,(1 - c_2\Theta\,)\right]^{1/2} d_p \tag{S4}$$

where c_1 and c_2 are dimensionless constants, $\Theta = S_g N_a$, is the coverage, S_g is the characteristic cross-section area of the vimentin aggregate and d_p is the layer thickness.

Hence, the layer thickness is given by

$$d_p = \left[c_1\Theta\,(1 - c_2\Theta\,)\right]^{-1/2} rms \tag{S5}$$

For a spherical aggregate one has c_1 =17/24 and c_2 = 50/51 [8]. For the tetramer these coefficients calculated by numerical integration are equal to c_1 =0.759 and c_2 = 1.01.

Using Eq.(S5) one can calculate that for the *rms* of 2.2 nm and Θ = 0.5, the vimentin layer thickness is equal to 5.1 nm.

3. Theoretical Analysis of Adsorption Kinetics

Protein molecule adsorption kinetics under diffusion and flow can be quantitatively interpreted in terms of the hybrid approach developed in Ref [9] where the following constitutive equation describing molecule transport through the adsorption layer of the thickness of δ_a

$$\frac{1}{S_g}\frac{d\Theta}{dt} = k_a\, n_i(\Theta)\; B(\Theta) - \frac{k_d}{S_g}\Theta = -\, j_b \qquad \text{(S6)}$$

where k_a, k_d are the adsorption and desorption constants, $n_i(\Theta)$ is the molecule (aggregate) concentration at the edge of this layer, $B(\Theta)$ is the blocking function, $j_b = -D_a \frac{\partial n}{\partial z}$ is the molecule flux at the edge of the adsorption layer, D_a is the molecule diffusion coefficient, n is the aggregate number concentration depending on the z coordinate perpendicular to the surface.

The absorption and desorption kinetic constants can be calculated *ab initio* if the interaction potential of the particle with the interface is known. For a deep energy minimum appearing at the distance δ_m and no energy barrier the desorption kinetic constant becomes negligible and the adsorption constant is given by [9]

$$k_a = \frac{D_a}{\delta_a[1+0.5\;\ln(\delta_a/\delta_m)]} \qquad \text{(S7)}$$

On the other hand, the blocking function can be calculated from stochastic coarse-grained modeling usually applying the random sequential adsorption (RSA) approach. As

discussed in Ref.[10] that for not too large coverage the blocking function can be approximated by the second order polynomial valid for particles of arbitrary shape

$$B(\Theta) = 1 - C_1\Theta + C_2\Theta^2 + 0(\Theta^3) \tag{S8}$$

For spherical or oblate spheroid aggregates adsorbing side on has $C_1 = 4$ and $C_2 = 6\sqrt{3}/\pi = 3.31$ [11]

A useful analytical expression was formulated in Ref.[11] allowing to interpolate the exact numerical results derived from RSA modeling for the entire range of coverage comprising the maximum coverage Θ_∞

$$B(\Theta) = \left[1 + 0.812\bar{\Theta} + 0.4258\left(\bar{\Theta}\right)^2 + 0.0716\left(\bar{\Theta}\right)^3\right]\left(1 - \bar{\Theta}\right)^3 \tag{S9}$$

where $\bar{\Theta} = \dfrac{\Theta}{\Theta_\infty}$.

For Θ approaching the maximum coverage, Eq.(S9) attains the limiting form:

$$B(\Theta) = 2.31\left(1 - \frac{\Theta}{\Theta_\infty}\right)^3 \tag{S10}$$

Eq.(6) serves as the kinetic boundary condition for the bulk transport equation derived within the framework of the convective diffusion theory in Ref.[12]. Because it is nonlinear in respect to the particle coverage, calculations of deposition kinetics under diffusion transport are only feasible using numerical methods by defining the coupling constant characterizing the ration of the bulk to surface transport resistances [9]

$$K = \frac{m_1}{\delta_a S_g c_b\ [1 + 0.5\ln(\delta_a / \delta_m)]} \sim \frac{2}{3\Phi_p[1 + 0.5\ln(\delta_a / \delta_m)]} \tag{S11}$$

where m_1 is the mass of a single molecule or aggregate, $c_b = n_b / m_1$ is the mass concentration of the protein in the solution n_b is the bulk number concentration and Φ_p is the volume fraction of the protein solution.

Eq.(S11) indicates that for low volume fractions, typical for vimentin adsorption experiments, the coupling constant becomes much larger than unity. In consequence the adsorption kinetics can be well approximated by the formula [9]

$$N_a = \Theta / S_g = 2 \left(\frac{D_a t}{\pi} \right)^{1/2} c_b / m_1 \qquad \text{(S12)}$$

where N_a is the surface concentration of protein molecules and D is their diffusion coefficient.

Eq.(S12) shows that protein adsorption kinetic from dilute solutions under diffusion regime is controlled by the bulk transfer and is linear in respect to the square root of time. The range of applicability of Eq.(S12) was estimated in this work performing numerical solution of Eq.(S6) coupled with the bulk transport equation applying a robust implicit finite-difference method. These calculations carried out for various bulk molecule concentrations and diffusion coefficients pertinent to vimentin aggregates confirmed the validity of Eq.(S12) for the range of time significantly exceeding the experimental adsorption time.

Therefore, Eq.(S12) was exploited in order to determine the aggregation number of vimentin molecules n_a by rearranging it to the following form

$$N_a / c_b = 2 \frac{Av}{n_a M_1} \left(\frac{D_a}{\pi} \right)^{1/2} t^{1/2} \qquad \text{(S13)}$$

where Av is the Avogadro constant M_1 is the monomer molar mass, thus $M_a = n_a\ M_1$ is the aggregate molar mass.

Because all variables appearing in Eq.(S13) are known, one can determine the aggregation number using the experimentally determined slope of N_a / c_b on the square root of the adsorption time $t^{1/2}$.

In contrast to the diffusion transport, in the case of convective-diffusion transport (QCM measurements), Eq.(S6) can be integrated to the following form [9]

$$\int_0^{\Theta} \frac{(K-1)B\left(\Theta'\right)+1}{K\,B\left(\Theta'\right)-K_d\Theta'}\, d\Theta' = S_g k_c (c_b / m_1) t \qquad \text{(S14)}$$

where, $K = k_a/k_c$ is the dimensionless coupling constant, k_c is the bulk mass transfer rate constant and $K_d = k_d/(S_g k_c n_b)$ is the dimensionless desorption constant.

For protein molecules the coupling constant becomes much larger than unity and Eq.(S14) in the case of no adsorption simplifies to the form

$$N_a = \Theta / S_g = k_c (c_b / m_1) t \qquad \text{(S15)}$$

This equation, which is valid for the coverage range below the maximum coverage, indicates that the protein coverage at the QCM sensor linearly increases with the bulk concentration and the adsorption time. Thus, by experimentally determining the dependence of N_a on t one can gain information about the stability of vimentin solutions under various physicochemical conditions.

4. Vimentin Corona Formation at Polymer Particles

Adsorption of vimentin at negatively charged SP particles, referred later on as corona formation in accordance with commonly used nomenclature [13-15] was carried out according to the following procedure. Initially equal volumes of the protein solution with the concentration ranging between 0.2 to 2 mg L^{-1} and the SP particle suspension of the concentration equal to 50

or 100 mg L^{-1} were mixed together over 15 min. After finishing an adsorption run, the electrophoretic mobility of the particles with the corona was measured by the LDV method and the corresponding zeta potential was calculated using the Smoluchowski formula. Primarily in these experiments, dependencies of the zeta potential of the particles on the vimentin bulk concentration were acquired.

It should be mentioned that the relaxation time of the corona formation can be calculated as

$$t_s \sim \frac{\delta_d^2}{\bar{D}} \qquad \text{(S16)}$$

where δ_d is the characteristic diffusion path, $\bar{D}_a$ is the mutual diffusion coefficient of the aggregate relative to the polymer particle. Because of a large size of the SP particles compared to the vimentin aggregate size, $\bar{D}_a$ is practically equal to the diffusion coefficient of the aggregate.

The diffusion path is given by the formula

$$\delta_d = \frac{1}{2} d_c \; [(\Phi_V / \Phi_{mx})^{-1/3} - 1] \qquad \text{(S17)}$$

where d_c is the diameter of polymer particles, Φ_V is their volume fraction and Φ_{mx} is the maximum volume fraction equal to 0.62 for a quasi-random packing of spheres.

Taking the vimentin aggregate diffusion coefficient of 5×10^{-11} $m^2 \cdot s^{-1}$, d_c = 820 nm and $\Phi_V = 10^{-4}$ one obtains from Eq.(S16) t_s = 1 s, which indicates that the corona formation time is very fast making kinetic investigations rather impractical. Thus, under the experimental conditions where the adsorption time was equal to 900 s, a full corona was formed characterized by the nominal coverage equal to

$$\Gamma = \left(\frac{\rho_p \, d_p}{6} \right) \frac{c_b}{c_p} \tag{S18}$$

where ρ_p and d_p, c_p are the polymer particle density, the diameter and the mass concentration, respectively.

Using Eq.(S18) experimental dependencies of the corona zeta potential on the coverage Γ were acquired at different pHs and NaCl concentrations. In Fig. S4 results obtained for pH 3.5 and 10 mM NaCl concentration are shown.

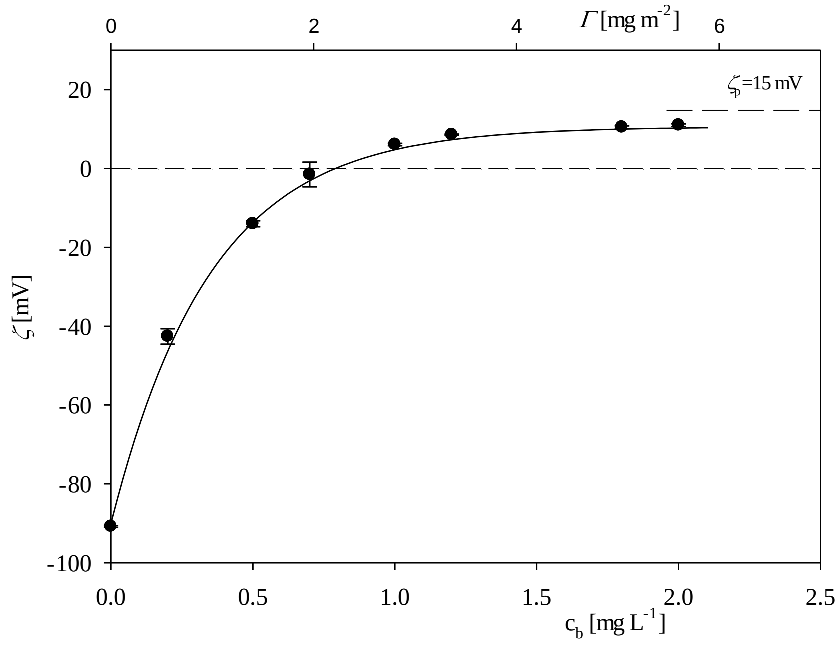

Fig. S1. The dependence of the zeta potential of vimentin at negatively charged SP polymer particles on the bulk protein concentration (lower horizontal axis), and nominal coverage calculated from Eq. (S18)(upper horizontal axis); adsorption conditions: pH 3.5, 10 mM NaCl concentration. The points denote experimental results obtained from the LDV measurements, the solid lines shows the theoretical results calculated from the electrokinetic model, Eq.(S19).

As can be seen, the initially negative zeta potential rapidly increases with the coverage and becomes positive for c_b larger than 0.7 mg L^{-1} (Γ larger than 2 mg m^{-2}). However, for still larger coverage, the change in the zeta potential become rather moderate and finally a plateau value equal to 10 mV is attained. The primary experimental data shown in Fig. S4 were theoretically interpreted in terms of the electrokinetic model discussed in Ref. [16] using the following expression for the zeta potential of protein corona at particles $\zeta_c(\Theta)$

$$\zeta_c(\Theta) = F_i(\Theta)\zeta_i + F_p(\Theta)\zeta_p \tag{S19}$$

where ζ_i is the zeta potential of bare polymer particles, ζ_p is the protein aggregate zeta potential in the bulk, and $F_i(\Theta), F_p(\Theta)$ are the dimensionless functions. F_i describes the damping of the flow at the interface by adsorbed molecules and the F_p function characterizes the contribution to the zeta potential stemming from the molecule surface charge.

The dimensionless coverage Θ is connected with the mass coverage by the dependence

$$\Theta = S_g\left(\frac{Av}{M_a}\right)\Gamma \tag{S20}$$

The hydrodynamic correction functions can be approximated for moderate thickness of the electric double-layers by the following expressions [17].

$$\begin{aligned} F_i(\Theta) &= e^{-C_i\Theta} \\ F_p(\Theta) &= a_p\Theta + b_p\left(1 - e^{-C_i\Theta}\right) \end{aligned} \tag{S21}$$

where the C_i, a_p and b_p coefficients for spherical particles layers assume the limiting values of 10.2, 0.202 and 0.618, respectively.

One can infer from Eq. (S21) that for a low protein coverage, the F_i function approaches unity and the F_p function vanishes. On the other hand, for the larger coverage of 0.5 the F_i function vanishes and the F_p function attains the value of $2^{-1/2} = 0.71$. Thus, using Eq.(S19) one can predict that the bulk zeta potential of vimentin aggregates can be calculated from the dependence

$$\zeta_p = \zeta_{c_\infty} / F_p = 2^{1/2} \zeta_{c_\infty} \qquad \text{(S22)}$$

where ζ_{c_∞} is the experimentally determined corona zeta potential in the limit of large protein coverage.

The bulk zeta potential determined in this way can be used to determine the net electrokinetic charge of aggregates Q_a as a function of pH from the following dependence [18]

$$Q_a = 2\pi\varepsilon d_H \left(1 + \kappa d_H\right) \zeta_p \qquad \text{(S23)}$$

where ε is the permittivity of the electrolyte, d_H is the hydrodynamic diameter of the aggregate and κ^{-1} is the electric double-layer thickness.

It is interesting to mention that Eq.(S19) can be inverted in to the following form yielding the protein corona coverage under *in situ* conditions

$$\Gamma = \frac{M_w}{AvS_g C_i} \ln \frac{\zeta_i - \zeta_{c_\infty}}{\zeta_c - \zeta_{c\infty}} \qquad \text{(S24)}$$

Acknowledgements:

This work was supported by the Statutory activity of the Jerzy Haber Institute of Catalysis and Surface Chemistry PAS and by the Research Grant of the National Science Center of Poland: UMO-2020/01/0/NZ6/00082 awarded to RB.

REFERENCES:

1. Morga, M., Adamczyk, Z., Gödrich, S., Oćwieja, M., Papastavrou, G., Monolayers of poly-L-lysine on mica – Electrokinetic charasteristics. *J. Colloid Interface Sci.* 456, 116-124, (2015).
2. Wasilewska, M., Adamczyk, Z., Sadowska, M., Boulmedais, F., Cieśla, M., Mechanisms of fibrinogen adsorption on silica sensors at various pHs: Experiment and theoretical modeling. *Langmuir* 35, 11275-11284, (2019).
3. Smoluchowski, M., Contribution a la theorie de l'endosmose electrique et de quelques phenomenes correlatifs. *Bull. Acad. Crac. Cl. Sci. Math Nat.* 182-199, (1903).
4. Wasilewska, M., Adamczyk, Z., Fibrinogen adsorption on mica studied by AFM and in situ streaming potential measurements. *Langmuir* 27, 686-696, (2011).
5. Dąbkowska, M., Adamczyk, Z., Ionic strength effect in HSA adsorption on mica determined by streaming potential measurements. *J. Colloid Interface Sci.* 366, 105-113, (2012).
6. Wasilewska, M., Adamczyk, Z., Pomorska, A., Nattich-Rak, M., Sadowska, M., Human serum albumin adsorption kinetics on silica: Influence of protein solution stability. *Langmuir*, 35, 2639-2648, (2019).
7. Bratek-Skicki, A., Sadowska, M., Maciejewska-Prończuk, J., Adamczyk, Z., Nanoparticle and bioparticle deposition kinetics: Quartz microbalance measurements. *Nanomaterials*, 11,145, (2021).

8. Morga, M., Nattich-Rak, M., Oćwieja, M., Adamczyk, Z., Gold substrates of controlled roughness and electrokinetic properties formed by nanoparticle deposition. *PCCP*, 21, 6535-6543, (2019).
9. Adamczyk, Z., Particles at interfaces: Interactions, deposition, structure, *Elsevier* (2017).
10. Evans, J.W., Random and cooperative sequential adsorption. *Rev Mod Phys*, 65, 1281-1329, (1993).
11. Schaaf, P., Talbot, J., Surface exclusion effects in adsorption processes. *J. Chem. Phys*. 91, 4401-4409, (1989).
12. Levich, V.G., Physicochemical hydrodynamics. *Englewood Cliffs, NY.: Prentice-Hall Inc* (1962).
13. Sheibani, S., Basu, K., Farnudi, A., Ashkarran, A., Ichikawa, M., Presley, J.F., Huy Buy, K., Reza Ejtehadi, M., Vali, H., Mahmoudi, M., Nanoscale characterization of the biomolecular corona by cryo-electron microscopy, cryo-electron tomography, and image simulation. *Nature Communications*, 12, 573, (2021).
14. Martinez-Negro, M., Gonzalez-Rubio, G., Aicart, E., Landfester, K., Guerrero-Martinez, A., Junquera, E., Insights into colloidal nanoparticle-protein corona interactions for nanomedicine applications. *Adv. Colloid Interface Sci.* 289, 102366, (2021).
15. Żeliszewska, P., Wasilewska, M., Cieśla, M., Adamczyk, Z., Deposition of polymer particles with fibrinogen corona at abiotic surfaces under flow conditions. *Molecules*, 26, 6299, (2021).
16. Adamczyk, Z., Sadlej, K., Wajnryb E., Nattich-Rak, M., Ekiel-Jeżewska, M.L., Bławzdziewicz, J., Streaming potential studies of colloid, polyelectrolyte and protein deposition, *Adv.Colloid Interface Sci*. 153, 1-29, (2010).

17. Ekiel-Jeżewska, M.L., Adamczyk, Z., Bławzdziewicz, J., Streaming current and effective ζ-potential for particle covered surfaces with random particle distribution. *J. Phys. Chem. C*, 123, 3517-3531, (2019).

18. Adamczyk, Z., Nattich-Rak, M., Dąbkowska, M., Kujda-Kruk, M., Albumin adsorption at solid substrates: A quest for a unified approach. *J. Colloid Interface Sci*. 514, 769-790, (2018).
